# Decline of influenza and respiratory syncytial virus detection in facility-based surveillance during the COVID-19 pandemic, South Africa, January to October 2020

**DOI:** 10.2807/1560-7917.ES.2021.26.29.2001600

**Published:** 2021-07-22

**Authors:** Stefano Tempia, Sibongile Walaza, Jinal N Bhiman, Meredith L McMorrow, Jocelyn Moyes, Thulisa Mkhencele, Susan Meiring, Vanessa Quan, Kate Bishop, Johanna M McAnerney, Anne von Gottberg, Nicole Wolter, Mignon Du Plessis, Florette K Treurnicht, Orienka Hellferscee, Halima Dawood, Fathima Naby, Ebrahim Variava, Comfort Siwele, Neydis Baute, Jeremy Nel, Gary Reubenson, Heather J Zar, Cheryl Cohen

**Affiliations:** 1 Influenza Division, Centers for Disease Control and Prevention, Atlanta, Georgia, United States; 2 MassGenics, Duluth, Georgia, United States; 3 School of Public Health, Faculty of Health Sciences, University of the Witwatersrand, Johannesburg, South Africa; 4 Centre for Respiratory Diseases and Meningitis, National Institute for Communicable Diseases of the National Health Laboratory Service, Johannesburg, South Africa; 5 Division of Public Health Surveillance and Response, National Institute for Communicable Diseases of the National Health Laboratory Service, Johannesburg, South Africa; 6 School of Pathology, Faculty of Health Sciences, University of the Witwatersrand, Johannesburg, South Africa; 7 Division of Virology, National Health Laboratory Service, Charlotte Maxeke Johannesburg Academic Hospital, Johannesburg, South Africa; 8 Department of Medicine, Greys Hospital, Pietermaritzburg, South Africa; 9 Caprisa, University of KwaZulu-Natal, Pietermaritzburg, South Africa; 10 Department of Paediatrics, Pietermaritzburg Metropolitan Hospital, Pietermaritzburg, South Africa; 11 Department of Paediatrics, University of KwaZulu Natal, Durban, South Africa; 12 Department of Medicine, Klerksdorp-Tshepong Hospital Complex, Klerksdorp, South Africa; 13 Department of Medicine, Faculty of Health Sciences, University of the Witwatersrand, Johannesburg, South Africa; 14 Perinatal HIV Research Unit, University of the Witwatersrand, Johannesburg, South Africa; 15 Department of Paediatrics, Matikwana Hospital, Mkhuhlu, South Africa; 16 Department of Paediatrics, Mapulaneng Hospital, Bushbuckridge, South Africa; 17 Department of Medicine, Helen Joseph Academic Hospital, University of the Witwatersrand, Johannesburg, South Africa; 18 Department of Paediatrics and Child Health, Rahima Moosa Mother and Child Hospital, Faculty of Health Sciences, University of the Witwatersrand, Johannesburg South Africa; 19 Department of Paediatrics & Child Health, Red Cross War Memorial Children’s Hospital, University of Cape Town, Cape Town, South Africa; 20 SA-MRC Unit on Child & Adolescent Health, University of Cape Town, Cape Town, South Africa

**Keywords:** COVID-19, influenza, respiratory syncytial virus, influenza-like illness, severe respiratory illness

## Abstract

**Background:**

In South Africa, COVID-19 control measures to prevent SARS-CoV-2 spread were initiated on 16 March 2020. Such measures may also impact the spread of other pathogens, including influenza virus and respiratory syncytial virus (RSV) with implications for future annual epidemics and expectations for the subsequent northern hemisphere winter.

**Methods:**

We assessed the detection of influenza and RSV through facility-based syndromic surveillance of adults and children with mild or severe respiratory illness in South Africa from January to October 2020, and compared this with surveillance data from 2013 to 2019.

**Results:**

Facility-based surveillance revealed a decline in influenza virus detection during the regular season compared with previous years. This was observed throughout the implementation of COVID-19 control measures. RSV detection decreased soon after the most stringent COVID-19 control measures commenced; however, an increase in RSV detection was observed after the typical season, following the re-opening of schools and the easing of measures.

**Conclusion:**

COVID-19 non-pharmaceutical interventions led to reduced circulation of influenza and RSV in South Africa. This has limited the country’s ability to provide influenza virus strains for the selection of the annual influenza vaccine. Delayed increases in RSV case numbers may reflect the easing of COVID-19 control measures. An increase in influenza virus detection was not observed, suggesting that the measures may have impacted the two pathogens differently. The impact that lowered and/or delayed influenza and RSV circulation in 2020 will have on the intensity and severity of subsequent annual epidemics is unknown and warrants close monitoring.

## Introduction

The severe acute respiratory syndrome coronavirus 2 (SARS-CoV-2) was first detected in South Africa on 5 March 2020 (week 10), and by the beginning of October there were over 690,000 laboratory-confirmed infections [1]. As part of the national response to the coronavirus disease (COVID-19) pandemic, the Government of South Africa declared a national state of disaster on 15 March 2020, after only 51 cases had been confirmed. Domestic and international travel restrictions and school closures were implemented on 16 March (week 12). A national lockdown with international and local travel restrictions, closure of all non-essential businesses and schools, and confinement of all persons in their residences was implemented from 27 March to 30 April (level 5 restrictions; weeks 13–18). This was followed by a phased easing of restrictions that allowed partial re-opening of selected businesses in May (level 4 restrictions; weeks 19–22). Additional businesses could operate from 1 June, when the progressive re-opening of schools was also implemented (level 3; weeks 23–33). From 17 August, schools were fully re-opened and the interprovincial travel ban was lifted (level 2 restrictions; weeks 34–38) and from 21 September all businesses could operate and a progressive resumption of international travel was implemented (level 1 restriction; week 39 onwards). Face mask use, hand hygiene and limits on mass gatherings and sport events were enforced at all restriction levels. All described measures may also impact the spread of other pathogens, including influenza and respiratory syncytial virus (RSV).

COVID-19 control measures in the northern hemisphere were mainly implemented towards the end of the 2019/20 winter, when influenza virus and RSV activity typically subsides; however, some countries documented an early decline in the detection of these viruses through surveillance [2-5]. At the time of writing, a reduction of influenza and RSV detection was described in some southern hemisphere countries during the 2020 winter (June–September); however, these studies are limited in the syndromic case definition for enrolment of patients (mainly influenza-like illness) and age strata included in the surveillance systems [2,6]. In addition, different control measures may have a different impact in different settings.

In this study, we assessed the detection of influenza and RSV through facility-based syndromic surveillance of adults and children with mild or severe respiratory illness in South Africa from January to October 2020 (up to 10 October 2020; weeks 1–41) and compared this with surveillance data from 2013 to 2019. In addition, we assessed the performance of the surveillance system during the same period.

## Methods

### Influenza-like illness and severe respiratory illness surveillance

From January 2013 to October 2020, surveillance for influenza and RSV was conducted among outpatients with influenza-like-illness (ILI) in four public health clinics in three of the nine provinces in South Africa and among inpatients with severe respiratory illness (SRI) in nine hospitals in five provinces. The structure of the system and the case definitions are outlined in Table 1 and have been previously described [7-9].

**Table 1:** Description of influenza-like illness and severe respiratory illness facility-based surveillance, South Africa, 2013–2020

Province	Number of surveillance sites	Period of surveillance	Surveillance case definition
**Influenza-like illness surveillance in public health clinics**			
North West	1	2013–2020	An outpatient of any age presenting with either temperature ≥ 38 °C or history of fever and cough for a duration of ≤ 10 days.
KwaZulu-Natal	1	2013–2020	
Western Cape	2	2018–2020	
**Severe respiratory illness surveillance in public hospitals**			
North West	2	2013–2020	A hospitalised person with symptoms of any duration who met age-specific clinical inclusion criteria: • Children aged 2 days to < 3 months: diagnosis of suspected sepsis or physician-diagnosed acute lower respiratory tract infection irrespective of signs and symptoms. • Children aged 3 months to < 5 years: physician-diagnosed acute lower respiratory tract infection, including bronchitis, bronchiolitis, pneumonia and pleural effusion. • Individuals aged ≥ 5 years: manifestation of acute lower respiratory tract infection with fever (≥ 38 °C) or history of fever and cough.
KwaZulu-Natal	1	2013–2020	
Mpumalanga	2	2013–2020	
Gauteng	2	2014–2020	
Western Cape	2	2015–2020	

All consenting patients meeting the case definition were eligible for enrolment and surveillance officers completed case report forms for enrolled patients. Respiratory specimens (i.e. nasopharyngeal aspirates or nasopharyngeal (NP) and oropharyngeal (OP) swabs from children aged < 5 years and NP and OP swabs from persons aged ≥ 5 years) were collected and tested for influenza and RSV using real-time reverse transcription PCR (rtRT-PCR) [10]. Influenza A virus-positive samples were further subtyped. From March to October 2020 all specimens collected through the ILI and SRI surveillance systems were further tested for SARS-CoV-2 using an rtRT-PCR assay targeting the SARS-CoV-2 envelope (E) gene [11].

### Data analysis

To assess the potential impact of the COVID-19 control measures on the enrolment of patients in the ILI and SRI surveillance systems we compared the cumulative weekly number of all enrolled patients with ILI or SRI (irrespective of SARS-CoV-2 positivity) in 2020 to the mean weekly values between 2013 and 2019. This analysis was implemented separately for patients with ILI and SRI, and in the following age strata: < 1, 1–4, 5–19, 20–44, 45–64 and ≥ 65 years. In addition, we excluded patients that tested positive for SARS-CoV-2 to assess the number of individuals enrolled in the surveillance systems with non-COVID-19–related illness.

Thereafter, we compared the weekly proportion of samples testing positive for influenza or RSV in 2020 to the mean weekly values in the period 2013 to 2019. The weekly value of the proportion positive for influenza and RSV were smoothed using a 3-week moving average. This analysis was implemented separately among patients with ILI or SRI.

### Ethical statement

The SRI protocol was approved by the University of the Witwatersrand Human Research Ethics Committee (HREC) and the University of KwaZulu-Natal Human Biomedical Research Ethics Committee (BREC) protocol numbers M081042 and BF157/08, respectively. The ILI protocol was approved by BREC protocol number BF080/12. This surveillance was deemed non-research by the United States (US) Centers for Disease Control and Prevention (non-research determination number: 2012–6197).

## Results

### Enrolment of patients with influenza-like illness or severe respiratory illness

In 2013 up to week 41 2020, a total of 10,439 patients with ILI were tested. In weeks 1–41 of 2020, the number of enrolled ILI cases was 1,183, which was in the range of the annual number (range: 622–1,504) of enrolled ILI cases in weeks 1 to 41 of 2013 to 2019. The number of enrolled SRI cases was 3,418 in weeks 1–41 of 2020, also in the range of the annual numbers (range: 1,634–4,005) in 2013 to 2019.

**Figure 1:**
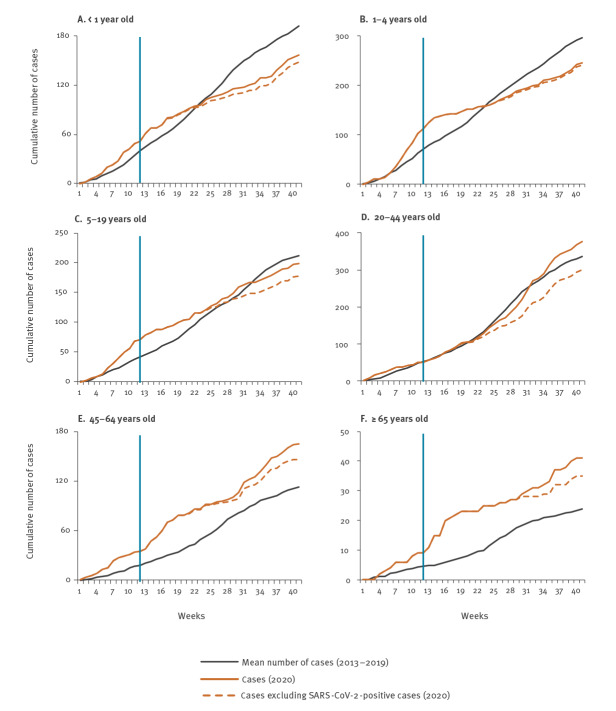
Cumulative weekly number of cases with influenza-like illness enrolled in facility-based surveillance, by age, South Africa, weeks 1–41 2013–2019 (mean) and 2020 (n=1,183)

COVID-19: corona virus disease; SARS-CoV-2: severe acute respiratory syndrome coronavirus 2.

In early 2020, the weekly number of enrolled ILI patients was greater than in the 2013 to 2019 period, but it became lower than in the earlier period in weeks 12–41 (Supplementary Figure S1, panel A). The decline in weeks 12–41 was predominantly related to fewer ILI enrolments aged 0–19 years (Figure 1, panels A–C).

The blue vertical line (week 12) indicates the start of the COVID-19 control measures in 2020.

**Figure 2:**
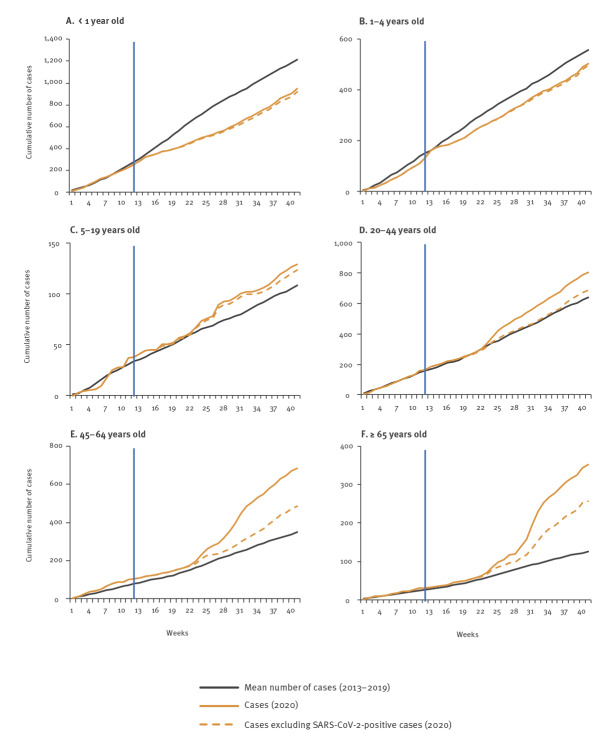
Cumulative weekly number of cases with severe respiratory illness enrolled in facility-based surveillance, by age, South Africa, weeks 1–41 2013–2019 (mean) and 2020 (n=3,418)

COVID-19: coronavirus disease; SARS-CoV-2: severe acute respiratory syndrome coronavirus 2.

The number of enrolled SRI patients in 2020 compared to the 2013 to 2019 period was similar for weeks 1–11, became lower in weeks 12–22 and then became higher in weeks 23–41 (Supplementary Figure S2, panel A). The decline is explained by a progressive reduction in enrolled SRI cases aged < 1 year and, to a lesser extent, those aged 1–4 years in weeks 14–41 (Figure 2, panels A–B). This was followed by an increase in enrolled SARS-CoV-2–positive SRI patients aged ≥ 20 years in weeks 23–41 (Figure 2, panels E–F).

The blue vertical line (week 12) indicates the start of the COVID-19 control measures in 2020.

### SARS-CoV-2 detection

From March to October 2020, SARS-CoV-2 was detected in 14.3% (135/946) and 16.1% (449/2,796) of specimens from ILI and SRI patients, respectively. The SARS-CoV-2 detection was highest in ILI patients aged 20–44 years (22.9%; 76/332) and in SRI patients aged 45–65 years (34.3%; 197/575). No co-infection with influenza and seven co-infections with RSV were detected. The weekly detection of SARS-CoV-2 among ILI and SRI patients is provided in Supplementary Figure S1, panel A and Supplementary Figure S2, panel A, respectively.

### Influenza virus detection

**Figure 3:**
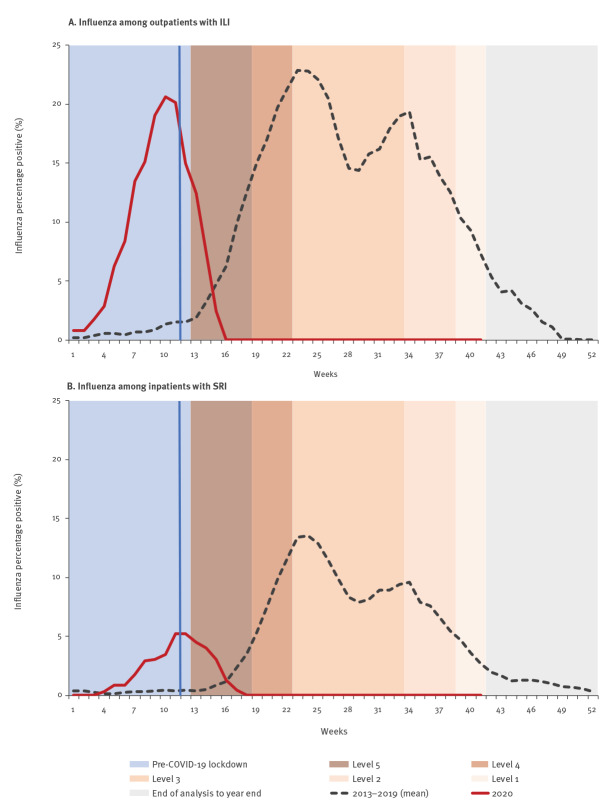
Percentage of influenza-positive patients (3-week moving average) enrolled in facility-based surveillance, South Africa, 2013–2019 (mean) and weeks 1–41 2020

COVID-19: coronavirus disease: ILI: influenza-like illness; SRI: severe respiratory infection.

From 2013 to 2019, influenza viruses were detected in 12.7% (1,175/9,256; annual range: 8.8–15.7%; range weeks 1–41: 10.3–16.4%) and 5.2% (1,319/25,118; annual range: 3.4–6.6%; range weeks 1–41: 4.3–7.4%) of specimens from ILI and SRI patients, respectively. Influenza virus detection was highest in ILI patients aged 5–19 years (21.6%; 380/1,761) and in SRI patients aged 1–4 years (8.1%; 399/4,945). The influenza peak (week with the highest proportion of influenza-positive cases) occurred between weeks 21–32 (median: week 23) in ILI surveillance and weeks 22–37 (median: week 24) in SRI surveillance. In 2020 (weeks 1–41), influenza viruses were detected in 4.4% (52/1,183) and 0.8% (26/3,418) of specimens from ILI and SRI cases, respectively. All influenza-positive samples in 2020 were detected during an out-of-season outbreak in the Western Cape Province (weeks 3–15; data not shown). No influenza viruses were detected in other provinces or after the Western Cape outbreak during the typical influenza season, as at week 41 2020 (Figure 3, panels A–B). The weekly number of influenza viruses detected between 2013 and week 41 2020 among ILI and SRI cases is provided in Supplementary Figure S1, panel B and Supplementary Figure S2, panel B, respectively.

The blue vertical line (week 12) indicates the start of the COVID-19 control measures in 2020, when domestic and international travel restrictions and school closures were implemented. The subsequent control measure levels comprised the following restrictions:

Level 5: international and local travel restrictions, closure of all non-essential businesses and schools, and country-wide stay-at-home orders.

Level 4: partial re-opening of selected businesses.

Level 3: additional re-opening of selected businesses and partial re-opening of schools.

Level 2: schools fully re-opened and interprovincial travel ban lifted.

Level 1: full re-opening of businesses and progressive resumption of international travel.

### Respiratory syncytial virus (RSV) detection

**Figure 4:**
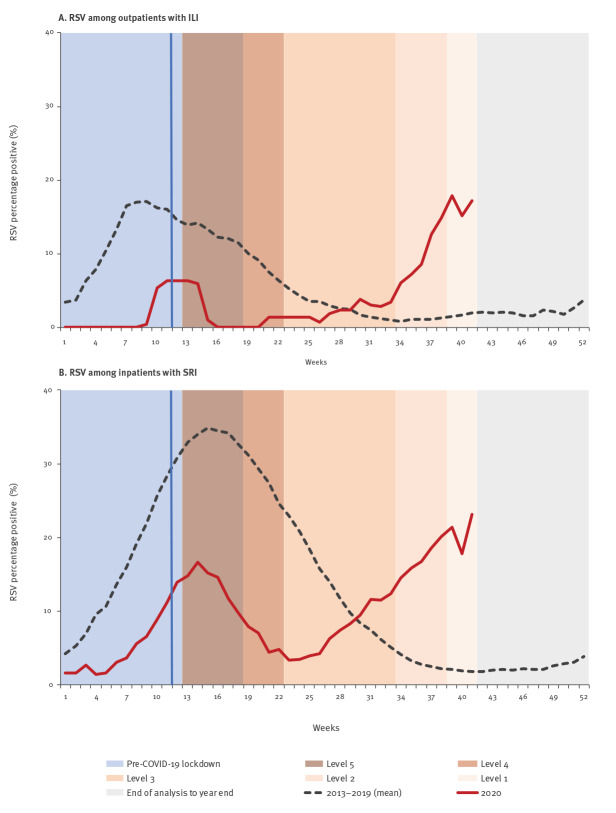
Respiratory syncytial virus percentage positive (3-week moving average) among patients enrolled in facility-based surveillance, South Africa, 2013–2019 (mean) and weeks 1–41 2020

COVID-19: coronavirus disease: ILI: influenza-like illness; SRI: severe respiratory infection.

From 2013 to 2019, RSV was detected in 6.0% (556/9,256; annual range: 4.2–8.4%; range weeks 1–41: 4.4–9.8%) and 15.4% (3,816/25,118; annual range: 9.8–17.8%; range weeks 1–41: 11.7–21.1%) of specimens from ILI and SRI patients, respectively. RSV detection was highest in infants aged < 1 year (ILI: 11.2%, 173/1,548; SRI: 30.5%, 3,057/10,032). The RSV peak (week with the highest proportion of RSV-positive cases) occurred between weeks 8–20 (median: week 12) in ILI surveillance and weeks 11–20 (median: week 16) in SRI surveillance. In 2020 (weeks: 1–41), RSV was detected in 4.1% (48/1,183) and 10.5% (358/3,418) of specimens from ILI and SRI patients, respectively. Following the implementation of COVID-19 control measures, an initial decline of RSV detection was observed in weeks 14–22, followed by an increase of RSV-positive cases from week 29 (Figure 4, panels A–B). The weekly number of RSV cases detected from 2013 to week 41 2020 among ILI and SRI cases is provided in Supplementary Figure S1, panel C and Supplementary Figure S2, panel C, respectively.

The blue vertical line (week 12) indicates the start of COVID-19 control measures in 2020, when domestic and international travel restrictions and school closures were implemented. The subsequent control measure levels comprised the following restrictions:

Level 5: international and local travel restrictions, closure of all non-essential businesses and schools, and country-wide stay-at-home orders.

Level 4: partial re-opening of selected businesses.

Level 3: additional re-opening of selected businesses and partial re-opening of schools.

Level 2: schools fully re-opened and interprovincial travel ban lifted.

Level 1: full reopening of businesses and progressive resumption of international travel.

## Discussion

Compared with previous seasons, in 2020 we observed a decline in the detection of influenza and RSV through facility-based surveillance. A reduced detection of influenza virus and RSV in 2020 has also been reported by a private laboratory group in South Africa [12]. A reduced detection of influenza viruses has also been observed in facility-based surveillance from other southern hemisphere countries—such as Australia, New Zealand, Chile and Argentina [6,13-16]—and significantly less influenza virus activity has been documented in the US and Japan during the COVID-19 pandemic (February−March 2020) [2,3].

Our surveillance systems remained fairly stable in terms of number of enrolled patients during weeks 12–41 2020, when the COVID-19 control measures were implemented; however, a decline in the enrolment of young children was observed, especially in SRI surveillance. This may reflect changes in healthcare seeking behaviour related to SARS-CoV-2 concerns; however, such changes are more likely to occur among patients with mild rather than severe illness. Lower RSV circulation following the implementation of the initial strict control measures (level 5)—as suggested by the initially low detection of RSV in young children during this period—may be an explanation for the observed decline of enrolled children with SRI. The numbers of COVID-19 cases in children were small, suggesting that replacement may not be responsible for the low detection of RSV in these patients. Movement restrictions, physical distancing, school closures and recommendations regarding face masks and hand hygiene may have lowered and/or delayed the spread of influenza and RSV. Nonetheless, low or delayed circulation of influenza and RSV due to other factors—such as temperature [17] or competition with SARS-CoV-2—cannot be excluded, but are thought to be unlikely.

An increase of RSV-positive cases was observed from week 29 (late spring/early summer), possibly because the COVID-19 control measures were eased, including the progressive re-opening of schools. The observed increase of RSV detection after week 29 occurred after the typical seasonal peak in South Africa, which occurs in late summer/early autumn (February to April). At the time of the analysis, it was unclear whether the observed increase in RSV detection following the easing of COVID-19 control measures signified an early start to the forthcoming RSV season, with sustained circulation into the typical season, or if this out-of-season RSV circulation would be followed by a decrease in RSV circulation before the typical RSV peak activity expected in early 2021.

An increase of influenza virus detection was not observed following the easing of COVID-19 control measures. The restrictions on international travel enforced in all five levels of the pandemic response may have played a role in the observed reduction of influenza and RSV detection by limiting external introduction. Such travel restrictions may have had a greater impact on the circulation of influenza viruses than of RSV, because of their more defined seasonality in South Africa. In contrast, RSV is detected throughout the year, with peak activity in the first half of the year, potentially sustaining pockets of local reservoirs. In addition, differences in the impact that COVID-19 control measures had on the spread of influenza vs RSV may be related to their transmissibility characteristics. The effective reproductive number (R) of seasonal influenza (R: 1.2–1.4) [18] is estimated to be lower than that of RSV (R: 1.7–2.1) [19], potentially resulting in COVID-19 control measures having a greater effect on influenza compared with RSV. Our results differ from those of Australia, where very limited circulation of both influenza and RSV was observed throughout the implementation of COVID-19 control measures [6]. This may be related to the implementation of different measures and/or different circulation or transmission patterns of influenza and RSV in the two countries.

It is estimated that, on average, over 11,000 influenza-associated deaths and over 6,000 RSV-associated deaths occur annually in South Africa [20]. The deaths caused by these two pathogens may have been partially prevented in 2020 by the implementation of COVID-19 control measures. The estimated mean annual influenza- and RSV-associated deaths provided above compare to preliminary estimates of 47,000 excess deaths as at week 41 2020, which may be directly or indirectly attributed to COVID-19 [21].

## Conclusions

The lack of detection of influenza viruses in South Africa during the typical influenza season, as at week 41 2020, has hindered the country’s ability to provide information on circulating viruses to inform the World Health Organization’s annual influenza vaccine strain selection [22] and to estimate the vaccine effectiveness of the 2020 southern hemisphere influenza vaccine in South Africa. A decline in the detection of influenza and RSV has been observed in facility-based surveillance. While the level of circulation of influenza viruses and RSV in the community remains unclear, the reduction was observed in both ILI and SRI surveillance programmes. Surveillance in community cohorts should assist in better understanding the population-level circulation of influenza and RSV. The impact of potentially lowered and/or delayed exposure and associated immunity to influenza and RSV in 2020 on the intensity and severity of subsequent annual epidemics is unknown and warrants close monitoring.

## Supplementary Data

286000 Supplementary Material
