# Supplementary material for: Decline of influenza and respiratory syncytial virus detection in facility-based surveillance during the COVID-19 pandemic, South Africa, January to October 2020
Source: Euro Surveill. 2021 Jul 22;26(29):2001600. doi: 10.2807/1560-7917.ES.2021.26.29.2001600 (PMC8299743; doi:10.2807/1560-7917.ES.2021.26.29.2001600)

Decline of Influenza and Respiratory Syncytial Virus Detection in Facility-Based Surveillance During the SARS-CoV-2 Outbreak in South Africa, January-October 2020 (Supplementary Material)

This supplementary material is hosted by *Eurosurveillance* as supporting information alongside the article "Decline of Influenza and Respiratory Syncytial Virus Detection in Facility-Based Surveillance During the SARS-CoV-2 Outbreak in South Africa, January-August 2020", on behalf of the authors, who remain responsible for the accuracy and appropriateness of the content. The same standards for ethics, copyright, attributions and permissions as for the article apply. Supplements are not edited by *Eurosurveillance* and the journal is not responsible for the maintenance of any links or email addresses provided therein.

Figure S1: Influenza-like illness (ILI) facility-based surveillance in South Africa, January 2013- October 2020 (week ending 10/10/2020). A: Weekly number of enrolled ILI cases. B: Weekly number of ILI cases testing positive for influenza. C: Weekly number of ILI cases testing positive for respiratory syncytial virus (RSV). The blue vertical line in 2020 (week 12) indicates the start of SARS-CoV-2 control measures.

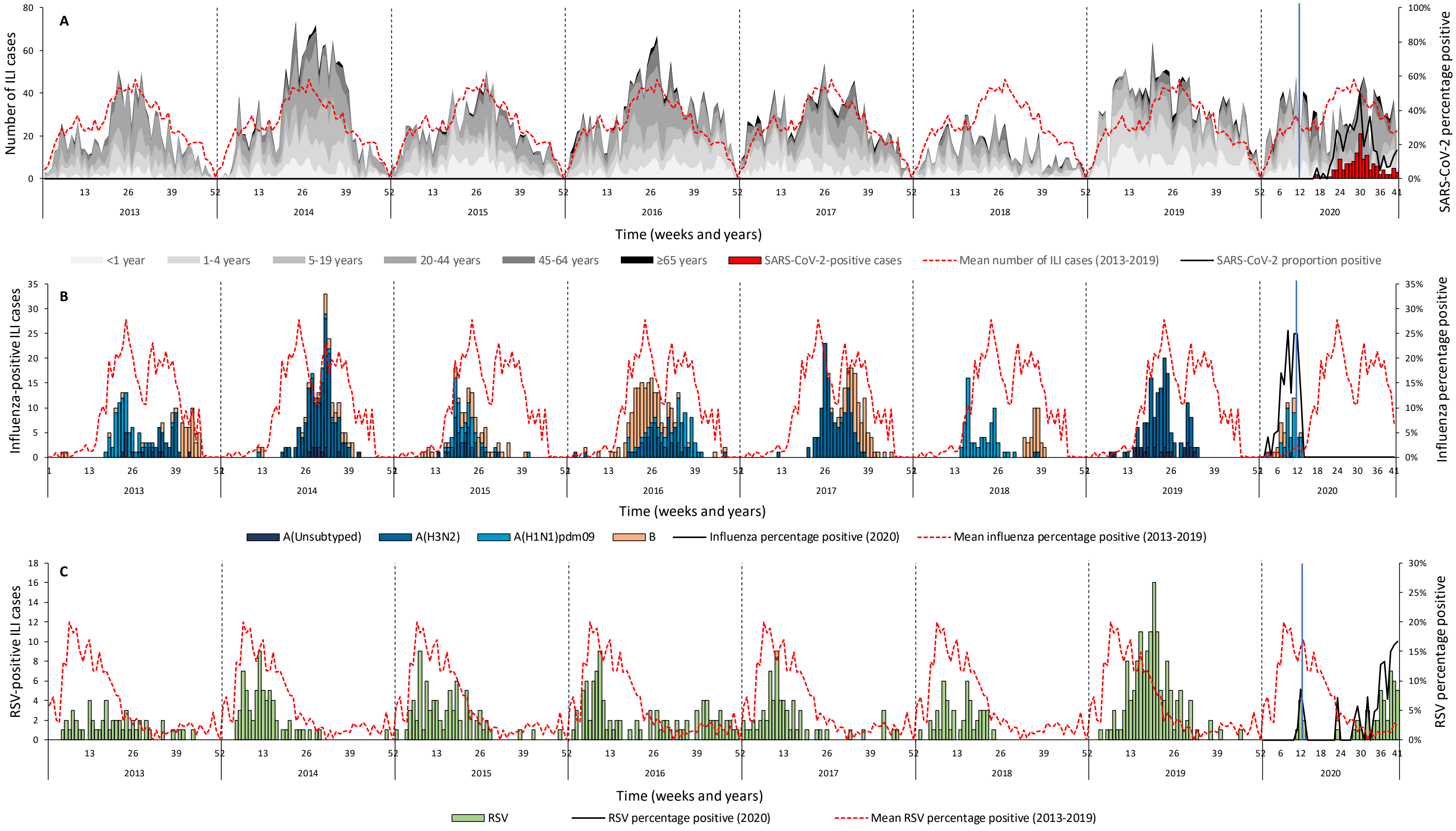

Figure S2: Severe respiratory illness (SRI) facility-based surveillance in South Africa, January 2013-October 2020 (week ending 10/10/2020). A: Weekly number of enrolled SRI cases. B: Weekly number of SRI cases testing positive for influenza. C: Weekly number of SRI cases testing positive for respiratory syncytial virus (RSV). The blue vertical line in 2020 (week 12) indicates the start of SARS-CoV-2 control measures.

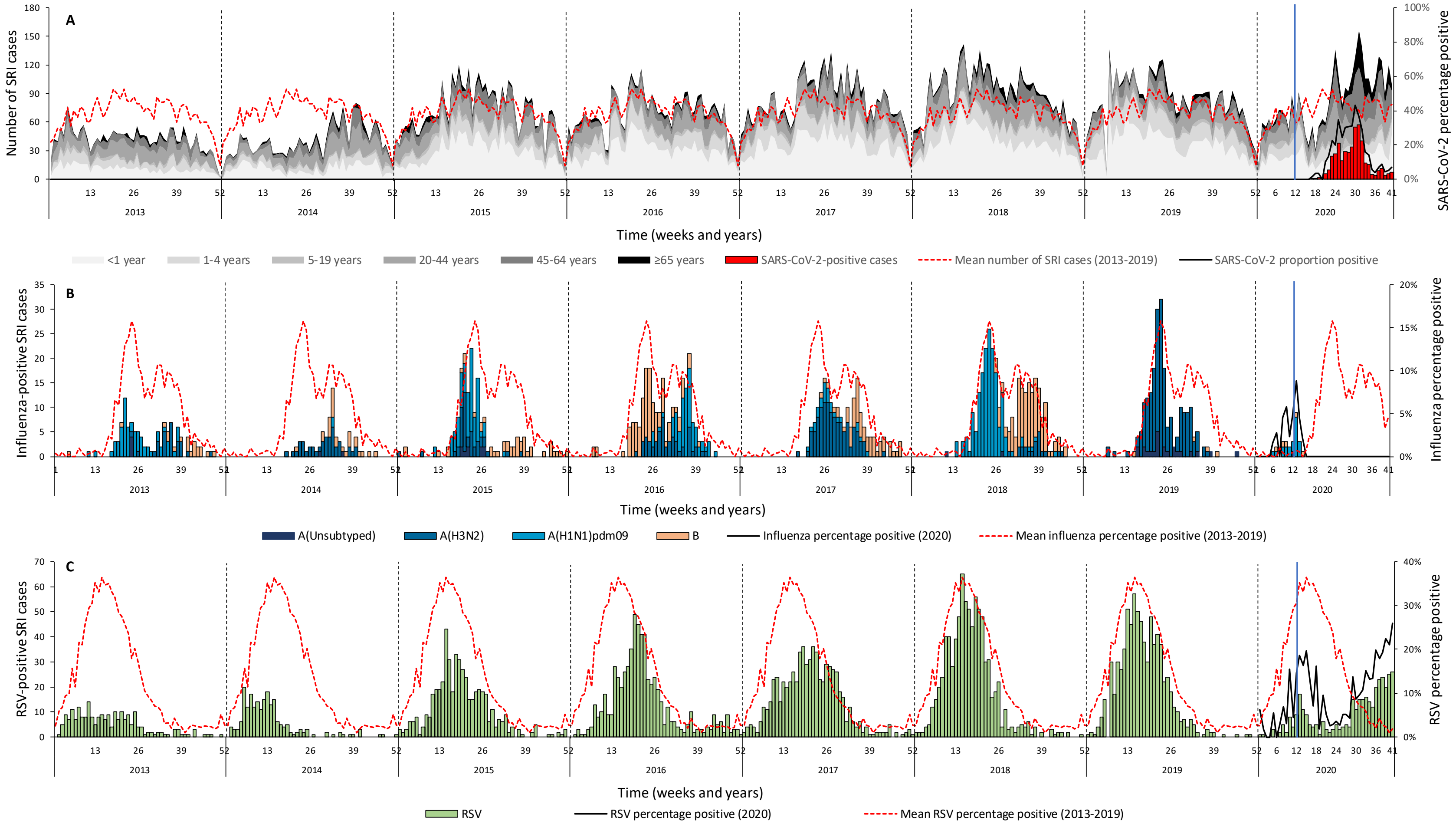

Supplement: Supplementary Material [file 20-01600_TEMPIA_supplementarymaterial.pdf]
